# On the accuracy of code complexity metrics: A neuroscience-based guideline for improvement

**DOI:** 10.3389/fnins.2022.1065366

**Published:** 2023-02-07

**Authors:** Gao Hao, Haytham Hijazi, João Durães, Júlio Medeiros, Ricardo Couceiro, Chan Tong Lam, César Teixeira, João Castelhano, Miguel Castelo Branco, Paulo Carvalho, Henrique Madeira

**Affiliations:** ^1^Faculty of Applied Sciences, Macao Polytechnic University, Macao, Macao SAR, China; ^2^Center for Informatics and Systems of the University of Coimbra (CISUC), University of Coimbra, Coimbra, Portugal; ^3^Center for Informatics and Systems of the University of Coimbra (CISUC), Polytechnic Institute of Coimbra, Coimbra, Portugal; ^4^Institute of Nuclear Science Applied to Health (ICNAS)/Coimbra Institute for Biomedical Imaging and Translational Research (CIBIT), University of Coimbra, Coimbra, Portugal

**Keywords:** code complexity metrics, code comprehension, EEG, cognitive load, mental effort, code refactoring, code constructs

## Abstract

Complexity is the key element of software quality. This article investigates the problem of measuring code complexity and discusses the results of a controlled experiment to compare different views and methods to measure code complexity. Participants (27 programmers) were asked to read and (try to) understand a set of programs, while the complexity of such programs is assessed through different methods and perspectives: (a) classic code complexity metrics such as McCabe and Halstead metrics, (b) cognitive complexity metrics based on scored code constructs, (c) cognitive complexity metrics from state-of-the-art tools such as SonarQube, (d) human-centered metrics relying on the direct assessment of programmers’ behavioral features (e.g., reading time, and revisits) using eye tracking, and (e) cognitive load/mental effort assessed using electroencephalography (EEG). The human-centered perspective was complemented by the subjective evaluation of participants on the mental effort required to understand the programs using the NASA Task Load Index (TLX). Additionally, the evaluation of the code complexity is measured at both the program level and, whenever possible, at the very low level of code constructs/code regions, to identify the actual code elements and the code context that may trigger a complexity surge in the programmers’ perception of code comprehension difficulty. The programmers’ cognitive load measured using EEG was used as a reference to evaluate how the different metrics can express the (human) difficulty in comprehending the code. Extensive experimental results show that popular metrics such as V(g) and the complexity metric from SonarSource tools deviate considerably from the programmers’ perception of code complexity and often do not show the expected monotonic behavior. The article summarizes the findings in a set of guidelines to improve existing code complexity metrics, particularly state-of-the-art metrics such as cognitive complexity from SonarSource tools.

## 1. Introduction

The high complexity of software, particularly code complexity, is traditionally considered the main contributing factor to software reliability issues (Rook, 1990). Complex code is hard to test, difficult to comprehend by programmers, and hence difficult to maintain. In addition to the intrinsic complexity of code structures, and the complexity related to the interconnection of components/artifacts, the size of the code is possibly the most expressive indicator of the very high levels of complexity of modern software. Today, many software systems easily reach millions of lines of code (LoC). For example, in the automotive industry, where a good share of the software is used for safety critical functions, a KPMG report from 2017 states that an “average car contains more than 150 million lines of code” (Silberg, 2017). The updated number for 2022 is certainly higher due to the constant increase in functionalities and sophistication of automotive software.

More LoC mean more bugs, as attested by the fact that the number of LoC is often used as the main metric to predict bug count in software products (Sandu et al., 2018). In fact, the same study reports field data from several real projects in the automotive area showing defect densities from 1 to 6 bugs per KLoC (Sandu et al., 2018), suggesting that the defect density remains quite significant. It is worth noting that the defect density reported in Sandu et al. (2018) is not drastically different from the defect rates per KLoC reported 25 years before in the seminal book from McConnell (1993), which indicated an industry average of 15 defects per KLoCs with a very large standard deviation. Although the defect density has been reduced due to advances in the software development processes and improved tools, the impressive increase in code size witnessed in the last decades has eroded the improvements in residual defect density. In other words, the problem of residual software defects remains the most persistent and difficult challenge of the software industry, and the major cause is the high complexity of software.

Measuring software complexity accurately is essential to control and minimize the negative effects of code complexity in software development. Software complexity metrics express quantitatively different aspects of the software and could be related to code, documentation, or even to the developers (Scalabrino et al., 2017). Metrics are heavily used in software engineering to guide the definition of test cases to achieve specific goals concerning test coverage (e.g., in using control flow and in data flow testing) (Ammann and Offutt, 2016), to predict software defects probability and estimate defect density (Moser et al., 2008; Huda et al., 2017), to determine the adequate component granularity in software architectures based on complexity thresholds (Herbold et al., 2011; Yamashita et al., 2016), to estimate reusability of software components (Papamichail et al., 2019), to control quality in continuous integration/continuous deployment (Fenton and Pfleeger, 2014; Garcia-Munoz et al., 2016), and to assess/predict how programmers comprehend code (Zuse, 1993; Sneed, 1995).

No matter the software development paradigm and specific flavor, software metrics play an important role in software development practices and have been the subject of intensive research. For example, a survey published in 2017 (Varela et al., 2017), focused only on code metrics, reported 226 studies published in a period of 5 years, from 2010 to 2015, proposing almost 300 different metrics, many of them related to code complexity. Another survey focused on using machine learning techniques for source code analysis (Sharma et al., 2021) reported 364 primary studies published between 2002 and 2021 including a large percentage of studies on topics such as code comprehension, refactoring, and code quality assessment. These examples give an idea of the research intensity and publications rate on the topic of software complexity and related metrics in recent years.

Despite this massive body of work, predicting/measuring software complexity in a way that accurately portrays the inherent complexity of software artifacts, as perceived by (human) software programmers, is still largely an open problem. Nevertheless, the human perceived complexity in understanding code is the measure that really matters for the development and maintenance of reliable software, as it is crucial to manage adequately important aspects such as software testability, modifiability, and reusability.

It is known that classic complexity metrics deviate considerably from human perceived complexity in code structures such as recursive or multi-threading programming, as in these cases the complexity is not in the code structures (usually compact) but in the recursive and/or parallel nature of the code. Several works have shown different aspects of this mismatch between complexity, as captured by code complexity metrics, and the real difficulties felt by programmers in comprehending code. For example, a relatively recent study (Ajami et al., 2017) including a group of 222 professional developers analyzed how programmers interpret code snippets with similar functionality but different structures and show significant differences (measured in speed and accuracy in comprehending code snippets) for the different structures, which clearly contradicts classic metrics such as cyclomatic complexity V(g) (McCabe, 1976) where all branching constructs are given the same weight. Other example (Jbara and Feitelson, 2017) shows that complexity metrics cannot capture context-sensitive aspects such as repeated code constructs that appear along the code.

Recent interdisciplinary works using biometric and neuroscience equipment (Couceiro et al., 2019; Medeiros et al., 2021; Peitek et al., 2021) provide neuroscientific evidence showing that classic complexity metrics such as V(g) do not capture well the difficulties experienced by programmers in comprehending code. These conclusions support the use of more elaborated cognitive complexity metrics such as the one used by state-of-the-art SonarSource tools (Campbell, 2017), but the question of whether such more elaborated metrics are accurate or not is still an open question. Since software refactoring is the main instrument to cope with code complexity in large-scale software projects, and refactoring is based on complexity metrics, it is of utmost importance to be sure that complexity metrics really represent code complexity as perceived by human programmers (the ones that develop, test, and maintain the code).

This article uses electroencephalography (EEG) to provide a reference for the assessment of the cognitive load of programmers while comprehending code. And it uses such reference to evaluate different views and methods to measure code complexity through a controlled experiment. A group of 27 software programmers (B.Sc. and M.Sc. students, and professional programmers) are asked to comprehend a set of programs, while the complexity of such programs is assessed through different methods including both code constructs and human-centered approaches to measure code complexity. Specifically, this study measures code complexity using the following methods:

a) Classic code metrics such as LoC, V(g), and Halstead metrics.
b) Cognitive complexity metrics based on scored code constructs.
c) Cognitive complexity metrics from state-of-the-art tools such as SonarSource tools.
d) Direct assessment of programmers’ behavioral features (e.g., reading time and revisits) using eye tracking.
e) Direct assessment of programmers’ cognitive load while comprehending the code using EEG, which has been proposed as a reference to measure cognitive complexity in code comprehension scenarios (Medeiros et al., 2019, 2021).

The human-centered perspective (d and e) is complemented by the subjective evaluation of participants on the complexity of the programs using the NASA Task Load Index (TLX) (NASA-TLX, 2020). Furthermore, the evaluation includes a dual approach of measuring code complexity at both the unit level and at the very low level of code constructs/code regions to expose the actual code elements that may trigger the perception of complexity from human perspective. The results are distilled as a set of guidelines to improve current methods and tools used to assess code complexity.

The structure of the article is as follow. Next section presents the related work, followed by the description of the controlled experiment design and setup in section “3 Controlled experiment design and setup.” Section “4 Results and discussion” discusses the results and proposes a set of guidelines on how to improve existing code complexity metrics. Section “5 Conclusion” concludes the article and briefly outlines the future work.

## 2. Related work

Code complexity has been extensively studied in the literature over the past decades for its importance in expressing software quality. Zuse (1991) defines code complexity as “the difficulty to maintain, change and understand software.” In the IEEE-Standard (1991) computer dictionary, the definition is “the degree to which a system or component has a design or implementation that is difficult to understand and verify.” Code complexity is the key element in predicting critical information about reliability, maintainability, and testability, among other software quality factors. It is, thus, essential to measure the code complexity and quantify it accurately to understand its effect on defect proneness and software quality (Sandu et al., 2018).

McCabe (1976) introduced the well-known cyclomatic complexity [V(g)] metric, which is the basis for the definition of test coverage in control flow testing techniques. V(g) measures the number of linearly independent paths in a code unit and expresses the difficulty in testing and maintaining the code (Ammar et al., 2001). The success of V(g) has led to the generalized use of this complexity metric as a measure of code understandability. Even today, V(g) is still used to control the complexity of code units, as a common industry practice is to refactor code units with V(g) higher than a given threshold [e.g., V(g) ≥ 10]. However, several works have shown the limitations of V(g) in expressing complexity from a programmers’ perspective (e.g., Ajami et al., 2017; Jbara and Feitelson, 2017; Couceiro et al., 2019; Peitek et al., 2021). For example, a clear limitation of V(g) is that it cannot distinguish between simple and complex condition statements. Additionally, case or switch statements that usually lead to repeated code patters that are easy to understand by programmers contribute to V(g) in a similar way as intricated (and difficult to understand) loop structures.

Halstead (1977) proposed a family of complexity metrics based on program operands and operators that attempt to express the difficulty, effort, programmers’ workload, and other measurable properties of software. Although the Halstead’s metrics are easy to calculate, and are particularly useful for data flow testing, they ignore the complexity of the control flow, as they are mainly focused on program data.

Overall, both V(g) and Halstead’s metrics neglect an essential factor: the human-centric perspective in expressing code complexity. In Kaur and Mishra (2019), the authors show that the human cognition involved in understanding or changing a code unit may hamper the software development because of the limited human cognitive resources. Developing, testing, and reviewing code are human intellectual and abstract processes. Therefore, mathematical models like those used in V(g) or Halstead’s metrics might be inadequate to assess the mental effort required to comprehend code.

Filling that gap, and trying to cope with modern programming languages structures, Wang and Shao (2003) and Wang (2006) introduced cognitive complexity (CC), which attempts to measure the functional complexity of the code in software design and code comprehension. Wang (2006) examined the cognitive weights of Basic Control Structures (BCS) and formulated what is called Cognitive Functional Size (CFS) to measure the software complexity from a cognitive perspective. BCS are defined as a collection of elements and flow controls to develop the code functional structure. These basic code constructs are classified under sequential, branch, iteration, embedded component, and concurrent structures, and each construct is assigned a score that represents the (expected) cognitive effort in comprehending such construct. The basic code construct scores are used to compute metrics of program cognitive complexity (Crasso et al., 2016; Jain and Satinderjit, 2019; Kaur and Mishra, 2019). However, the different flavors of this metric consider that code complexity increase in a linear way, which deviates significantly from the human perception of code complexity, where the saturation effect has been observed (i.e., if a subject considers a code unit very complex, adding more complexity to the code does not change the human perception as a very complex code) (Couceiro et al., 2019).

A new metric of cognitive complexity emerged in 2017 from SonarSource tools and is currently one of the most popular complexity metrics used by the software industry (Campbell, 2017). Although this SonarSource metric was also intentionally designed to measure code understandability (and is also called cognitive complexity), its approach is considerably different from the idea proposed by Wang and Shao (2003) and Wang (2006) and subsequent papers (Crasso et al., 2016; Jain and Satinderjit, 2019; Kaur and Mishra, 2019) that rely on scoring basic code constructs. The cognitive complexity metric disseminated by SonarSource tools is an attempt to improve code refactoring decision based on V(g). It ignores structures that allow multiple code statements to be shorthanded into one statement, increments by one for each break in the linear flow, and increments when flow-breaking structures are nested, trying to express the complexity programmers may feel in handling the code (Campbell, 2017).

SonarSource metric is in fact the most popular code complexity metric today, but, to the best of our knowledge, its accuracy in the assessment of code complexity from programmers’ perspective has not been evaluated so far using neurophysiological measures of cognitive load and mental effort extracted using EEG while programmers are comprehending code, as we do in the current article.

Numerous efforts have been made to investigate code comprehension and understandability and its relationship with programmers’ cognitive load while performing tasks on code [e.g., answering comprehension questions (Rilling and Klemola, 2003), understanding different source code patterns (Fakhoury et al., 2018), or bug detection in code reviews (Hijazi et al., 2022)]. Scalabrino et al. (2021) conducted a study to calculate the correlation between 121 complexity metrics and proxy variables for code understanding gathered in an experiment. They showed that none of the examined metrics [including V(g) and LoC] could capture the essence of code understandability. Other empirical studies have investigated the correlation between code complexity metrics and code understanding using classic approaches based on surveys and calibrated questions (e.g., Kasto and Whalley, 2013) or, more recently, medical imaging equipment to effectively measure mental effort and cognitive load while reading and understanding code (Peitek et al., 2018; Castelhano et al., 2019, 2021). Although some of these studies report correlations between the metrics and the subjects’ performance on tasks related to code comprehension, a general conclusion from available studies is that classic code complexity metrics failed to capture the (human) difficulty in comprehending code in many code patterns.

Recent code comprehension measurement trends have begun using physiological measures captured from the software programmers while reading and (attempting to) understand code. Physiological measures, such as HRV, EEG, or eye-tracker measures (Müller and Fritz, 2016; Couceiro et al., 2019), have shown a successful capacity to quantify software programmers’ cognitive, mental workload, and comprehension levels. This new area was coined as NeuroSE (neuro software engineering) in a recent comprehensive survey (Weber et al., 2021) where the authors proposed the term NeuroSE to “describe a research field in software engineering (SE) that makes use of neurophysiological methods and knowledge better to understand the software development” (Weber et al., 2021).

There are two main strands of research in this context: the use of information captured from the Central Nervous System (CNS) and the application of surrogate information captured mainly from the Autonomic Nervous System (ANS) activity. The latter exhibit significant potential for real-life implementation due to the less intrusive technologies required (e.g., smart watches and eye trackers), whereas the former is more accurate (hence more adequate for research purposes) but less interesting for developing solutions for real-life deployment since they rely on more intrusive technologies such as functional magnetic resonance imaging (fMRI) and EEG.

So far, most research in NeuroSE targeting code comprehension is focused on brain activity measurements using fMRI and EEG due to their higher accuracy (Weber et al., 2021). In fact, it has been shown that EEG can be used to accurately identify programmers’ cognitive load associated with understanding code of varying complexity (Medeiros et al., 2021). In Duraisingam et al. (2017) and Ishida and Uwano (2019), EEG features taken from several brain regions are applied to perform a thorough analysis of task difficulty level for program comprehension. Furthermore, there is clear evidence that the complexity of the code induces mental effort that can be assessed using EEG (Medeiros et al., 2019). In Lee et al. (2016), Crk and Kluthe (2014), and Crk et al. (2016), EEG-based feature analysis was applied to classify expertise level of software programmers. Indeed, it is observed that cognitive performance in code comprehension tasks will differ since expertise-related differences in subject performance can be assessed using EEG indications of working memory during code comprehension tasks. Information fusion of eye movement and EEG has been applied to predict programmer expertise and task difficulty (Lee et al., 2018).

Although fMRI and EEG-based approaches can measure programmers’ mental effort and cognitive load while comprehending software code (Weber et al., 2021), these approaches cannot be used in real software development settings because of their inherent intrusiveness (e.g., EEG would require the programmers to wear an EEG cap). Thus, it is vital to evaluate existing code complexity metrics to assure that code complexity inferred automatically from code features, as current practice in the software industry, really represents the complexity of the code as perceived by programmers. This is precisely the goal of this article that compares classical complexity metrics, cognitive complexity based on code constructs, and SonarSource cognitive complexity with physiological (EEG) and behavioral measures that represent the mental effort of software programmers associated with code comprehension tasks.

## 3. Controlled experiment design and setup

In general terms, the controlled experiment performed in this work is a code comprehension study. The programs used in the code comprehension tasks have been designed to show different levels of complexity according to classic complexity metrics. A group of participants, software programmers, were asked to perform three code comprehension trials. Each trial consists of a control task and a program comprehension task. The experimental setup included an EEG quick cap and EEG amplifier to acquire the EEG signals, and an eye tracker to allow us to know where each participant was looking at during the code comprehension tasks. Although the EEG and the eye tracker are separated devices, the data streams of both devices are synchronized using a common time base. The eye tracker was additionally used to measure the time each participant spent in each region of the code and to count the number of revisits to specific parts of the code of each program, as the process of comprehending code normally includes several iterations. At the end of each trial, the participant answered to a small number of questions designed to assess the degree of comprehension of each program. Additionally, each participant also filled out a NASA-TLX (2020) survey to indicate his/her subjective assessment of the code comprehension tasks.

The next subsections describe the elements of the controlled experiment and setup. All the relevant data (experiment protocol, programs used, code regions, and anonymized data on the individual participants) is available in this link^1^ as Supplementary material for this article.

### 3.1. Overview of the experiment protocol

**FIGURE 1:**
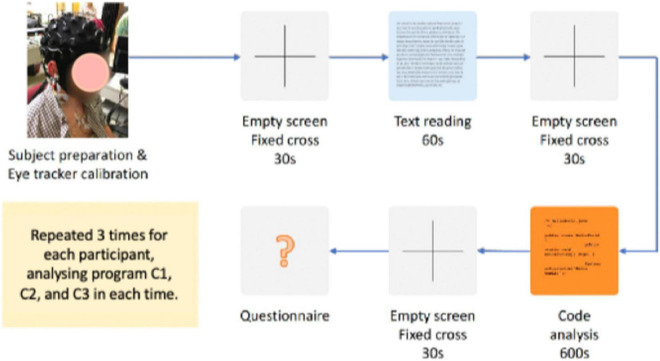
Sequence of steps for each trial of program comprehension.

Figure 1 shows a diagram representing the experiment protocol. For each participant, the experiment started with some preparatory steps such as the installation of the EEG quick cap, eye tracker calibration, recap of the trial steps, and answer to any questions/doubts from the participant. The participant was acquainted with the setup and informed about the sequence of steps of the trial and maximum time allocated to each task.

The steps of each trial were the following:

a) Fixation cross screen: an empty screen with a cross in the middle, shown for 30 s, used as baseline interval to separate tasks.
b) Control task: reading a narrative/descriptive text written in the native language of the participants for 60 s. This task was designed to create an activity that does not require significant effort, providing us with a cognitive load baseline for the EEG observations.
c) Code comprehension task: comprehension of a program in Java for a maximum allowed time of 600 s (10 min). Participants were allowed to stop earlier if they think that they have fully understood the program.
d) Questionnaire: survey with two questions about the program (“What does the program do?” and “How does the program work?”) to assess how well each participant understood each program, followed by the NASA-TLX (2020) survey to assess the subjective impression of each participant about the code comprehension task, particularly metal effort, time pressure, and level of discomfort felt during the execution of the task. The participants’ answers to these questions were evaluated by the authors of this article and scored in a scale from 0 to 6 (same scale as NASA-TLX). The final score representing how well each participant understood each program is the average of the scores assigned to the answer provided for the two questions.

Each participant performed three trials, covering a different program in each trial. The participants did not have any previous knowledge about the programs used in the code comprehension tasks and have not received any hint about the complexity of each program to avoid bias. The programs were shown to the participants always in the same order (i.e., no randomization) to assure all participants had the same conditions for the program comprehension tasks. Since the focus of our study is on the participants (i.e., evaluation of participants’ cognitive load and comparison with the code metrics scores), and not on the classification of the programs, we gave priority to assuring the same conditions to all participants.

The experiment design and protocol have been approved by the Ethics Committee of the Faculty of Medicine of the University of Coimbra, in accordance with the Declaration of Helsinki. All the participants involved in the experiment have signed an informed consent and all the data collected was anonymized to assure full privacy of participants.

### 3.2. Programs and code regions of analysis

The programs used in the controlled experiment are three Java programs (named as C1, C2, and C3) specifically designed to study code comprehension and programmers’ cognitive load induced by the code comprehension tasks. To avoid extraneous elements that may bias the perception of complexity, all the programs followed three general requirements:

• Do not require specific domain-level knowledge from the participants; the algorithms involved are generic computation tasks.
• Do not use obscure or uncommonly hard-to-read syntax.
• Do not involve many libraries that are not part of the language itself.

We designed the programs to cover different levels of code complexity to better understand what triggers difficulty when trying to comprehend code. The programs were evaluated by a set of software development experts to rank them based on their perceived difficulty. Program C1 is the easiest to understand, program C2 has a medium difficulty, and program C3 is the hardest. All the programs are relatively small to meet the experiment time limits and avoid the effects of mental fatigue of the participants that could skew our results. C2 and C3 have similar size (a bit more than 40 LoC). Program C1 is organized in two functions (methods in a Java class) referred here as units of code; program C2 comprises 3 units, and program C3 is a single unit. Table 1 summarizes the features of the programs.

**TABLE 1:** Key features of the programs used in the study.

Program	Expected complexity	V(g)	LoC	Number of units	Goal
C1	Easy	5 + 1	15	2	Counts the number of elements in an array that fall within a given interval.
C2	Medium	4 + 7 + 1	45	3	Computes multiplication using classic weighted digit algorithm.
C3	Difficult	22	43	1	Searches 3 dimensional objects in a 3 dimensions space.

We partitioned the source code of the programs into small regions to better identify and study the aspects of the source code that may be related to difficulty when trying to comprehend the code. This partitioning is aligned with the notion that programs and units of code (e.g., functions) are typically too large to be a single focus of attention by programmers at a given time. Instead, programmers usually focus on a specific area of code at a time and process it as a single unit of attention. Boundaries of such regions are typically defined by programming language constructs, in particular those that cause control flow branching (e.g., if-conditions and loops), or by the high-level logical nature within the algorithm they serve (e.g., a region of variable initialization, a region of parameter checking, a region of calculations, etc.). Having the source code of the programs partitioned into small regions allows us to analyze in detail the aspects of programming language specific to each region and their relationship with the cognitive load experienced by participants when trying to comprehend that code.

The strategy used for partitioning is straightforward and can be implemented into an automated tool if needed. There are two or more levels of regions: top-level regions and nested regions. Top-level regions are immediately obtainable from syntactically frontiers of the source code (as described next), and nested regions are parts of top-level regions or other nested regions.

Top-level regions are defined as follows:

• Each function (or “method”) in a program is a top-level region, provided that the program has more than one function. Otherwise, the program is a single unit.
• Inside a single unit program or a function, top-level regions are defined by identifying loop constructs (for, while) that act as logical separators of the code at algorithmic level. The entire loop and its inner (subordinated) instructions are one top-level region, the code before the loop is another top-level region, and the code after the loop is another top-level region.
• In the case the code unit has an initial set of instructions that correspond to variable declarations of 3 or more lines, then there is a logical separation of code from the programmer’s point of view that act as a natural separator just as a top-tier loop construct: variable declaration then the algorithm steps. In this case, the declarations are one top-level region, and the code is one or more top-level regions (depending on having loop constructs). The number of lines of variable declarations may be adjusted. In this study, 3 lines were considered as the minimum to justify an independent region.
• Top-level regions do not overlap with one-another.

Sub-regions result from partitioning top-level regions (or other sub-regions, depending on the nesting). They are obtaining by recursively applying to the region being partitioned the method that is used to partition a program function into top-level regions, with small differences due to the smaller code-size:

• The outermost loop constructs act as separators to define the new level of sub-regions.
• Conditional branching can also be used as separators in the same manner as loop constructs: at this level of detail of depth inside the algorithm of the code, branching is as relevant as loops. Only outermost branching is considered.
• Blocks of lines of variable declarations can also constitute sub-regions.
• Sub-regions can be further partitioned into sub-regions.

Table 2 presents an example of partitioning for program C2. It is worth noting that participants were not aware of this partitioning as it is a conceptual tool for our analysis without any visible traces in the code presented to them.

**TABLE 2:** Region partitions example for C2 program.

Regions		Program C2
**C2.A**		01 private static byte[] getInts(String digs) { 02 byte[] result = new byte[digs.length()]; 03 for (int i = 0; i < digs.length(); i++) { 04 char c = digs.charAt(i); 05 if (c < ‘0’ | | c > ‘9’) { 06 throw new IllegalArgumentException(“Invalid string” + c 07 + “at position” + i); 08 } 09 result[digs.length() - 1 - i] = (byte) (c - ‘0’); 10 } 11 return result; 12 }
**C2.B**		13 public static String getResult(String num1, String num2) { 14 byte[] left = getInts(num1); 15 byte[] right = getInts(num2); 16 byte[] result = new byte[left.length + right.length];
	**C2.C.1**	17 for (int rightPos = 0; rightPos < right.length; rightPos++) { 18 byte rightDigit = right[rightPos]; 19 byte temp = 0;
**C2.C**	**C2.C.2**	20 for (int leftPos = 0; leftPos < left.length; leftPos++) { 21 temp + = result[leftPos + rightPos]; 22 temp + = rightDigit * left[leftPos]; 23 result[leftPos + rightPos] = (byte) (temp % 10); 24 temp / = 10; 25 }
	**C2.C.3**	26 int destPos = rightPos + left.length; 27 while (temp ! = 0) { 28 temp + = result[destPos] & 0xFFFFFFFFL; 29 result[destPos] = (byte) (temp % 10); 30 temp / = 10; 31 destPos++; 32 } 33 }
**C2.D**		34 StringBuilder stringResultBuilder = new StringBuilder(result.length); 35 for (int i = result.length - 1; i > = 0; i–) { 36 byte digit = result[i]; 37 if (digit ! = 0 | | stringResultBuilder.length() > 0) { 38 stringResultBuilder.append((char) (digit + ‘0’)); 39 } 40 } 41 return stringResultBuilder.toString(); 42 }
**C2.E**		43 public static void main(String[] args) { 44 System.out.println(getResult(“1234”,“56789”)); 45 }

We did not predefine a target for the number of regions no preferred region size. Code was partitioned as long as no high-level language constructs were broken, and the resulting sub-regions maintained a self-contained meaning. This resulted in several levels on nested regions. We included all levels of regions in our study and not only the innermost regions. A total of 29 code regions were defined in the three programs: C1 has only 3 top-level regions, C2 has 5 top-level regions and 3 inner regions, and C3 has 3 top-level regions and 15 inner regions.

### 3.3. Participants

The recruitment of participants started with a call for participation asking for participants with experience in Java programming language. The selected group of 27 participants include B.Sc. and M.Sc. students, researchers, and software professionals. Out of the 27 participants, 21 are males and 6 are females, reflecting the gender unbalance among software developers. The age range is from 19 to 42 years old, with an average age of 24.4 years and a standard deviation of 6.12 years.

The screening process for selection of the participants was mainly focused on the assessment of the programming experience of the participants through an interview that included a survey with questions about the number of years in software development, the size of Java software projects in which they have worked on, and the frequency of Java programming tasks. A final group of 27 programmers were divided in two groups (for the analysis of results), according to their acquaintance to the Java language, as declared in the survey. Since the participants’ answers in the survey are subjective, we also considered the participants’ performance in program comprehension tasks to fine-tune the clustering of the participants in the following groups:

• Ordinary programmers: participants with Java programming experience (have completed at least one course on Java programming in their bachelor’s degree), although some of them do not program in Java frequently (19 participants).
• Proficient programmers: participants with good skills in Java programming, with at least 3 years of Java experience, and frequently involved in programming tasks in Java in the last 3 years (8 participants).

### 3.4. Electroencephalography

The EEG signal was acquired through the Neuroscan SynAmps 2 amplifier from Compumedics at a sampling rate of 1,000 Hz and utilizing 64 channels placed through an EEG quick cap connected to the amplifier through the EEG head-box. The EEG headbox module is connected to the acquisition computer that collects the signals from the sensors. The placement of the EEG electrodes in the scalp used the well-known international 10-10 system (Graimann et al., 2010).

Electroencephalography measures the electrical activity of the brain and can be an essential tool to collect direct measurements of the participants’ brain activity while trying to comprehend the software. The goal is to assess the participants’ cognitive load induced by the code comprehension tasks. The assumption is that complex code will induce higher levels of cognitive load in participants when compared to the cognitive load associated with the comprehension of simple code.

The EEG signal can be divided into many waveforms based on frequency, amplitude, and spatial distributions. Table 3 shows the five main frequency bands (Delta, Theta, Alpha, Beta, and Gamma), which are the ones most frequently utilized (Malmivuo and Plonsey, 1995) to extract features for cognitive load assessment.

**TABLE 3:** Typical analyzed EEG frequency bands [adapted from Medeiros et al. (2021)].

Name	Frequency range	Associated state of brain
Delta (δ)	<4 Hz	Unconscious/deep sleep
Theta (θ)	4–8 Hz	Conscious/imagination/memory
Alpha (α)	8–13 Hz	Conscious/relaxed mental activity
Beta (β)	13–30 Hz	Conscious/emotional/focused
Gamma (γ)	>30 Hz	Conscious/high mental activity

The next paragraphs briefly describe the preprocessing of EEG signals and the features extraction.

#### 3.4.1. Preprocessing

In order to get a reliable analysis of the neural signals, the recorded EEG data must first be preprocessed. We followed the typical EEG preprocessing pipeline, starting with filtering the raw EEG data, which is followed by inspection and interpolation of channels with lower quality signal, re-referencing and, finally, using Blind Source Separation (BSS) for further artifact removal such as involuntary ocular movements (eye blinking and microsaccades). This preprocessing step was done using the open-source toolbox EEGLAB (Delorme and Makeig, 2004). The specific procedure is shown in Table 4.

**TABLE 4:** The procedure of EEG signal processing.

Step Number	Name	Tool	Objective
1	Filtering	High-pass filter with a cut-off frequency at 1 Hz	Remove the DC component and slow wave drifts.
		Low-pass filter with a cut-off frequency of 90 Hz	The upper limit of the frequency band of interest for the analysis.
		Notch filter at 50 Hz	Remove the powerline interference.
2	Channels spatial interpolation	Spherical spline interpolation algorithm from Perrin et al., 1989	Remove and replace flat or noisy channels with interpolated signals based on the data from the remaining channels.
3	Re-referencing	Average reference	Eliminate some common noise to all channels and reduce lateralization bias.
4	Blind source separation	Independent component analysis (ICA)	Additional artifact removal (ocular, muscle, cardiac, and residual artifacts).

After cleaning the EEG data, a handcrafted feature engineering approach was followed by exploring reported features in the literature on cognitive load. This stage consists of feature extraction, normalization, transformation, and scaling to achieve the final cognitive load measure calculation.

#### 3.4.2. Feature extraction

To extract the proper EEG features for each code region, we explored the features reported in the paper (Malmivuo and Plonsey, 1995) that were proposed to accurately identify programmers’ cognitive load associated with understanding code with different levels of complexity. The explored EEG features fEEG1 and fEEG2 were extracted from the electrode F2 and electrode PZ in the 10-10 placement system (Graimann et al., 2010), respectively, and are described as follows:

##### fEEG1 (index 1 of the task engagement indexes)

This is one of the indexes proposed firstly by Pope et al. (1995) and is currently widely used as a measure of engagement during tasks (Freeman et al., 2004; Lei, 2011). Specifically, index 1 is the ratio between Theta, Alpha, and Beta power bands as presented in the following equation:


(1) fEEG1=(θ+β)/α

##### fEEG2 (power ratio between Theta and Alpha bands)

The power ratios between frequency bands have been explored to minimize the variability effects between subjects and in Malmivuo and Plonsey (1995), the ratio between the Theta and Alpha band was found to be as one of the most relevant features and is described by the equation below:


(2) fEEG2=θ/α

The absolute power of the Theta (4–8 Hz), Alpha (8–13 Hz), and Beta (13–30 Hz) frequency bands was obtained by computing the area under the power spectrum density (PSD) curve. The PSD was calculated by squaring the absolute value of the fast Fourier transform of the clean EEG signal. Finally, we computed the ratios between the frequency bands of interest to obtain the two features to be explored in this study.

#### 3.4.3. Feature normalization

After feature extraction, to reduce the inter-subject and intra-subject variability, we normalized the feature values of the code task under analysis with respect to the control task. The final extracted features values represent then the variation of the features on the code task in comparison to the control task:


(3) $$△\mathrm{Feature}{\mathrm{Code}}_{C}\left(k\right)=\frac{\mathrm{Feature}{\mathrm{Code}}_{C}\left(\text{k}\right)-\bar{\text{Control}}\left(k\right)}{\bar{\text{Control}}\left(k\right)}$$

where *Feature Code*_*C*_ (*k*) is the vector of the values of the feature *k* from the code *C* being normalized by $\bar{Control}\left(k\right)$, which is the average value of the feature *k* in the control task.

#### 3.4.4. Feature transformation

To capture potentially rapidly changing cognitive load dynamics during code inspection, four parameters were computed from each feature for each analysis window considered. These parameters are maximum, minimum, mean, and standard deviation.

#### 3.4.5. Feature scaling

Before proceeding to the analysis, the final transformed EEG features were normalized considering min-max normalization to eliminate scale dependencies among the different participants.


(4) $$x^{\prime }=\left(x-\min\left(x\right)\right)/\left(\max\left(x\right)-\min\left(x\right)\right)$$

### 3.5. Eye tracking

The eye tracker device is a remote binocular eye tracking (SMI RED) system (SMI-SensoMotoric Instruments, Germany), with a sampling rate of 120 Hz. The tracker has a reported gaze position accuracy of 0.4° and a spatial resolution of 0.05°. The participants sat between 60 and 70 cm away from a 22-in flat screen with a resolution of 1,680 × 1,050 pixels. The system compensates for head movements within a 50 cm × 30 cm (at 65 cm distance), allowing the participants to look at the screen in a naturalistic manner. A 9-point calibration procedure with a fixation cross was performed before each task.

The stream of timestamped data with the coordinates of gaze points produced by the eye tracker were synchronized with the EEG through a common time base, in order to allow the association of the code region where the subject was looking at a given moment with the corresponding EEG features. The eye-tracker was also utilized to measure the time each participant spent reading each program and the time spent in each code region of the programs. Since the process of reading and comprehending code is normally iterative, we have also measured the number of revisits of participants to each code region. The participants’ reading time and, more specifically, the distribution of the reading time along the code regions, combined with the number of revisits to each code region, provide a relevant indication of the complexity perceived by the subject while reading and understanding the code.

### 3.6. NASA-TLX

In our experiment, we used NASA-TLX as an additional source of information to indicate the perceived difficulty of reading and comprehending the three programs. NASA-TLX (2020) provides the participant’s subjective workload assessment of a given task considering a multidimensional rating process based primarily on a weighted average of ratings from six dimensions: (a) Mental Demand; (b) Physical Demand; (c) Temporal Demand; (d) Performance; (e) Effort; and (f) Frustration. Since NASA-TLX is quite general, we considered only the dimensions that make sense in code comprehension tasks (e.g., we did not consider Physical Demand).

### 3.7. Cognitive load measurement

**FIGURE 2:**
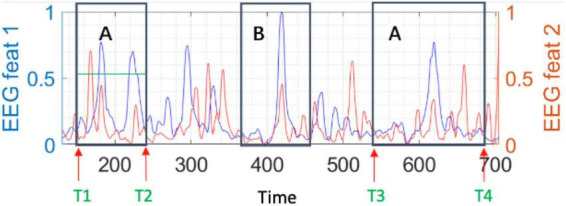
An illustrative example of the two EEG features (fEEG1 and fEEG2) values over the time for a given code comprehension task, with two specific regions of interest A and B.

To obtain a measure to be used as reference in our study, we assessed the programmer’s cognitive load directly through the combination of the two extracted EEG features (fEEG1 and fEEG2) and calculated the sum of the area under the curve of both features while the programmer is looking at a specific code region for a given duration (see Figure 2).

In the illustrative example in Figure 2, we can observe the two extracted EEG features (fEEG1 and fEEG2) and the time intervals corresponding to the participant’s reading of specific code regions (A and B). The programmer’s cognitive load while comprehending the code of a given region for a given amount of time (note that we know where and when the participant is looking at through the eye tracker data) is represented by the area under the curve of the two EEG features during that period. The larger the area under the curve, the higher the cognitive load associated with the participant’s comprehension of that code region. This way, both the amplitude of the EEG features and the duration of the time window spent by the participant on comprehending the code region will determine the measured cognitive load of the participant for such code region.

Since participants may read (i.e., visit) each code region more than one time when comprehending a specific code region (e.g., region A in Figure 2 is visited twice), we measure the participant’s cognitive load for the code region as the sum of the cognitive load measured in each visit. For example, for code region A, the participant’s cognitive load is the sum of the areas under the curve of both features from the instance T1 to the instance T2 (first visit) and from T3 to T4 (second visit).

The numeric value for the cognitive load of each participant while he/she is trying to comprehend a given code region considers as a reference (i.e., lower bound) the cognitive load measured for that participant during the control task (reading a simple narrative/descriptive text written in the native language of the participant) and the scale normalization described above. The higher the numeric value, the higher the cognitive load of the participant.

## 4. Results and discussion

This section starts with the presentation of global results in section “4.1 NASA-TLX results, code comprehension performance, and cognitive load (EEG),” showing the subjective perception of complexity (measured using NASA-TLX) and the cognitive load (measured using EEG) for the three programs, as well as the participants’ performance in comprehending each program. Section “4.2 Code complexity metrics results” presents the detailed results for all the metrics considered in the study and the corresponding cognitive load measured by EEG, covering both the entire programs and the detailed analysis at the code region-level within each program. Section “4.3 Guidelines to improve code complexity metrics” summarizes the most relevant guidelines to improve code complexity metrics and Section “4.4 Limitations and threats to validity” discusses limitations and threats to the validity of our study. Along the entire section, to facilitate reading, we include key research questions, and the corresponding answers and observations.

### 4.1. NASA-TLX results, code comprehension performance, and cognitive load (EEG)

NASA Task Load Index is a key instrument to measure the mental effort felt by participants, according to their own subjective points of view. Its relevance in this work is two-fold. First, NASA-TLX results allow us to evaluate our assumptions concerning task design into low (C1), medium (C2), and high code complexity (C3). Second, and even more important, these results corroborate our proposal that cognitive load measured using EEG (Medeiros et al., 2021) can be used to analyze how well current code complexity metrics (i.e., the ones evaluated in this study) are good indicators for the programmers’ difficulty in comprehending code or not.

**FIGURE 3:**
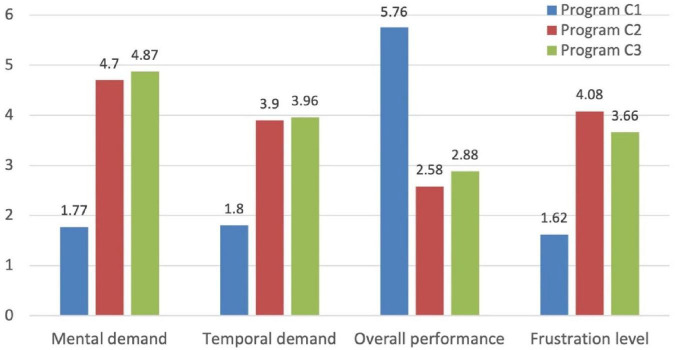
NASA-TLX average results considering all participants.

Figure 3 shows the results for the four NASA-TLX dimensions considered in this study. The results are the average of the scores (from 0 to 6, being 6 the subjective maximum scored by participants) provided by all participants to each question of the NASA-TLX survey.

The participants’ subjective perception of their mental demand/effort in understanding the code of the 3 programs shows that C1 was considered much easier (i.e., required less mental effort) than C2 and C3, while C2 and C3 were regarded by the participants at a similar level of complexity. As shown previously in Table 1, C3 has a single unit with cyclomatic complexity V(g) of 22 (very complex) and C2 includes 3 units with V(g) of 4, 7, and 1. Despite having very different values of cyclomatic complexity, the participants considered both programs at a similar level of complexity.

The Mann–Whitney U test of H_0_: “the results of mental demand for programs C2 and C3 have the same distribution,” considering the results of the 27 participants, shows *p* = 0.522, which means we cannot reject H_0_ (i.e., participants consider that C2 and C3 required the same mental demand).

*RQ1 – Is there a saturation effect in the programmers’ perception of code complexity?*

*Participants’ perception revealed a code complexity saturation effect as they considered that the mental effort required to understand both C2 and C3 programs is similar. More code complexity (at least from a cyclomatic perspective) in C3 does not translate into a perception of more mental effort, suggesting that participants have reached their own limit of mental effort.*

The perception expressed by participants dividing the programs in two groups, simple (C1) and complex (C2 and C3), contradicts our initial assumption for the design of the three programs: according to the participants’ perspective, we do not have a program representing medium complexity code. Nevertheless, the study is not significantly affected by the lack of medium complexity task because the analysis of the different metrics is also done at the code region level, and we have a large variety of regions of different sizes and complexity (a total of 29 code regions, considering top level regions and inner regions).

The results for the other NASA-TLX dimensions are consistent with the results obtained for mental effort. The perception on how well each participant has understood the programs (Figure 3, third group of results from the left) shows that participants are almost sure that they have understood C1 (an answer of 6 means that the participant was totally sure) and they were quite unsure about what programs C2 and C3 do and how they work. The results for the temporal demand in the execution of the tasks (the code comprehension tasks had a maximum of 10 min for all programs) and the frustration level are also quite consistent with the observation that participants found C1 easy to understand and C2 and C3 are comparably difficult.

Table 5 shows the average results for the participant perception of mental effort (from NASA-TLX), the cognitive load measured using EEG, reading time, and the participants performance in comprehending each program. As mentioned before, the participants’ performance in comprehending the code was graded by reviewing the participants’ answers to the questions “What does this code do?” and “How does it work?”

**TABLE 5:** Average global results: performance, cognitive load, and reading time (95% confidence).

	Mental effort (NASA-TLX)	Cognitive load (EEG)	Reading time (seconds)	Performance (comprehension)
**All Participants**				
Program C1	1.77 (± 0.25)	21.95 (± 6.86)	80.76 (± 20.95)	5.45 (± 0.23)
Program C2	4.70 (± 0.38)	93.95 (± 14.52)	504.38 (± 31.27)	2.60 (± 0.50)
Program C3	4.87 (± 0.39)	123.03 (± 34.49)	457.14 (± 107.76)	2.73 (± 0.59)
**Ordinary programmers**				
Program C1	1.95 (± 0.34)	21.63 (± 6.71)	76.45 (± 17.80)	5.13 (± 0.28)
Program C2	4.74 (± 0.42)	90.58 (± 29.70)	495.24 (± 56.52)	1.97 (± 0.22)
Program C3	4.95 (± 0.47)	108.9 (± 71.88)	418.23 (± 220.24)	2.13 (± 0.54)
**Proficient programmers**				
Program C1	1.45 (± 0.35)	22.70 (± 18.96)	88.31 (± 55.36)	6.00 (± 0.00)
Program C2	4.64 (± 0.86)	97.13 (± 17.46)	512.38 (± 42.42)	3.68 (± 1.11)
Program C3	4.73 (± 0.80)	141.86 (± 33.55)	509.03 (± 93.70)	3.75 (± 1.17)

The next paragraphs discuss the most relevant observations from the results shown in Table 5. Note that the values obtained for the cognitive load measured using EEG are related (i.e., normalized) to the control task (a reference task requiring very low cognitive load). The higher the value obtained for cognitive load (EEG), the higher the cognitive effort required compared to the control task.

The cognitive load measured using EEG is consistent with the subjective perception of mental effort declared by participants. In fact, the average cognitive load (EEG) for C1 is relatively low, while the values for C2 and C3 are much higher than the one observed for C1. Additionally, the cognitive load (EEG) for C2 and C3 are similar. This is the same pattern observed for the mental effort in NASA-TLX. The Spearman correlation coefficient (*r*_*s*_) calculated for the scores obtained for the mental effort (NASA-TLX) and cognitive load (EEG) for all participants in the three programs is *r*_*s*_ = 0.829, with a corresponding *p* < 0.0001, which indicates a high correlation between the cognitive load measured using EEG and the subjective perception of mental effort declared by the participants.

*RQ2 – Can EEG be a reference to assess the difficulty perceived by programmers in comprehending source code?*

*(a) Results support the assumption that cognitive load (EEG) measured in our experiment represents well the difficulty programmers may perceive in comprehending code, which is precisely the (human) perception of code complexity that ideal code complexity metrics should capture. Thus, cognitive load measured using EEG is a good yardstick to compare complexity metrics, as we do in this* article.

Concerning participants’ performance in the code comprehension tasks (right hand column in Table 5), the average performance of the C1 program (the one requiring low mental effort/cognitive load) is high (5.45 on a 0 to 6 scale). Actually, all the proficient programmers fully understood program C1 (scored 6.0). For the programs causing higher mental effort/cognitive load (C2 and C3), the average performance in comprehending the code drops significantly. This drop in the performance is, as expected, less evident for the experienced programmers, when compared to ordinary programmers. For ordinary programmers the drop in performance from program C1 to program C2 is 3.16 (more than 50% of the scale range), while for proficient programmers the decrease in performance is only 2.32 (38%). Globally, when the programmers’ cognitive load (EEG) is high (i.e., revealing that the participants really required a significant cognitive effort to understand the code), we observe that the performance in comprehending the code decreases.

The Mann–Whitney U test of H_0_: “the cognitive load (EEG) measured while participants were comprehending C1 is not related to the participants’ performance in understanding program C1” shows *p* < 0.001, which means we can reject H_0_. The tests for C2 and C3 produce similar results. Since the test is directional, we can state that the higher the measured cognitive load (EEG) the lower the participant’s performance in comprehending the code.

*(b) This observation also supports our assumption of using cognitive load (measured by EEG) in this experiment as the reference to express the difficulty perceived by programmers in comprehending code, as when the program requires a high cognitive load, the performance in comprehending the code drops significantly.*

Obviously, we are not suggesting that cognitive load can be used as a predictor of performance in comprehending a program in real life, as many other factors are involved (e.g., the person could not try seriously to understand the program, which will result in a low cognitive load, or the person may be mentally busy with other issues while trying to understand a given code). However, in the context of this controlled experiment high levels of cognitive load were caused by high complexity in the code, which resulted in low performance for the complex programs.

Reading time (second column from the right in Table 5) also shows a clear difference for program C1 when compared to both C2 and C3. All the three code comprehension tasks had a maximum allocated time of 10 min (600 s), but participants were allowed to stop earlier. In fact, participants needed much less than 10 min to understand program C1 (average of 80.76 s with a large confidence interval). On the contrary, participants used almost the entire time slot of 10 min for programs C2 and C3.

The Spearman correlation coefficient (*r*_*s*_) calculated for the reading time and cognitive load (EEG) measured for all 27 participants in the three programs is *r*_*s*_ = 0.9857, with a corresponding *p* < 0.001, which indicates a high correlation between the reading time of each program and the cognitive load measured using EEG.

Although reading time may seem a good metric to indicate programmers’ difficulty in comprehending code, a more detailed analysis reveals obvious limitations of reading time as an indicator of code complexity. Since C3 is clearly very complex [V(g) = 22 in 43 LoC], we noticed that some participants gave up and stopped trying to understand the code before the end of the 10 min slot, which justifies an average reading time for C3 a bit lower than the average reading time for C2. This is also the reason why proficient programmers show reading times longer than ordinary programmers, as proficient programmers tried to understand C2 and C3 almost until the end of the 10 min window (they did not give up).

*RQ3 – Is reading time a reliable indicator of code comprehension difficulty?*

*(a) The observation of eye tracking data for individual participants confirmed that even when the reading time is long (i.e., the participant used the entire 10 min slot), some participants showed a reading pattern that suggests they were engaged and trying to understand the code for some time and gave up at a given moment, and simply kept looking at the code in a random way. This shows that reading time might not be a reliable indicator of code complexity.*

**FIGURE 4:**
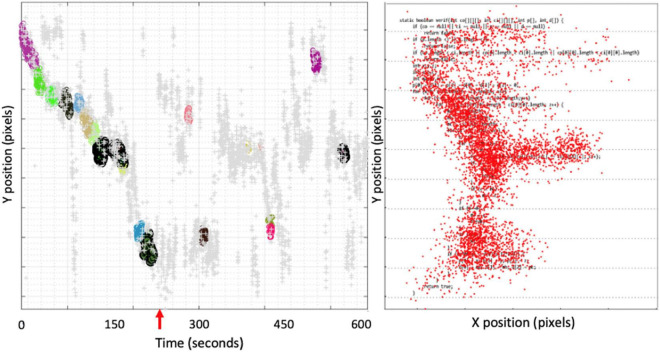
Eye tracker data for Participant 2 in the comprehension task for program C2.

Figure 4 shows an example of eye tracker data to illustrate the disengagement of the participant from the code comprehension task (an example for Participant 2 during code comprehension of C2). The right side of Figure 4 shows the heat map of the geometrical distribution of gaze points superimposed on code during the whole window of 10 min. On the left side of Figure 4, the gaze points are clustered using Y-coordinate (i.e., only consider code lines and ignore the *X*-coordinate inside each code line). The clustered gaze points are represented along the time, and the different clusters (in different colors) represent reading velocity. The clustered gaze points on the left are aligned with the code lines on the right and represent the moments when the participant read the corresponding code line. Gaze points marked in gray correspond to high velocity code reading (i.e., not the type of reading that represents a serious effort to understand the code). The rationale of for using code reading velocity was that complex sections of code are expected to exhibit lower reading velocities, while less complex code lines should have higher reading velocity.

Figure 4 shows that Participant 2 started reading the code and did a full detailed read until the end of the code, which took around 220 s (point marked by the red arrow). After that point on, the participant continued looking at the code at high reading velocity (gray gaze points), with occasional gaze clusters distributed without a consistent reading pattern. In other words, although the reading time of this participant was close to 600 s, the code comprehension period was limited to the first 220 s.

*(b) Reading time must be carefully considered when assessing code comprehension difficulty in controlled experiments, as it can easily assume a confounding behavior (nevertheless, reading time is often used in the literature to assess programmers’ code comprehension difficulty).*

The fact that proficient programmers tried harder and used longer engaged reading times, when compared to ordinary programmers, explains why we did not observe low values of cognitive load for proficient programmers. In fact, if a programmer is proficient, we would expect a lower value of cognitive load in program comprehension tasks, when compared to ordinary programmers. However, we did not observe a large difference in cognitive load between proficient and ordinary programmers due to the tendency of proficient programmers to perform a thorough reading that resulted in longer average reading time (and consequently, a higher accumulated cognitive load).

### 4.2. Code complexity metrics results

Table 6 shows a global view of the results for all the metrics studied in our work considering the three programs and their structure (units). As indicated in Table 1, C1 is composed of a function and the main unit, C2 has two functions and the main unit, and C3 is a monolithic program with a single unit. To keep consistency with the analysis at the more detailed level of code regions presented further on, Table 6 also indicates the identifiers of the code regions between parentheses. The metrics considered include (LoC) and V(g) (McCabe, 1976), a group of five metrics based on data and program operands (Halstead, 1977), cognitive complexity based on weighting basic control structures (CC-BCS) (Wang, 2006; Kaur and Mishra, 2019), cognitive complexity from SonarSource (CC-Sonar) (Campbell, 2017), and cognitive load measured using EEG (used as reference).

**TABLE 6:** Global results for all the code complexity metrics considered in the study.

Program	LoC	V(g)	Volume (HVol)	Length (HLth)	Effort (HEff)	Nb. of operands	Difficulty (HDif)	CC-BCS	CC-Sonar	Cognitive load (EEG)		
										All	Ordinary	Proficient
C1.function (C1.AB)	10	5	222.9	93	4,557.2	23	20.4	192	5	14.35	14.99	13.33
C1.main (C1.C)	5	1	208.9	43	1,319.3	24	6.3	9	0	7.60	6.64	9.38
C2.function1 (C2.A)	12	4	489.9	90	9,650.5	39	20.6	1,550	4	25.54	21.85	28.77
C2.function2 (C2.BCD)	30	7	1,295.0	224	62,116.5	107	48.0	6,608	9	66.16	67.30	65.29
C2.main (C2.E)	3	1	81.75	20	286.1	10	3.5	8	0	2.25	1.43	3.06
C3	43	22	2,147.6	395	162,705.7	–	–	4,379,690	54	123.03	108.90	141.86

A first observation from the results in Table 6 is focused on the less complex code units. Although the main units of C1 and C2 scores relatively high in the Halstead data-oriented metrics, they have V(g) of 1 and CC-Sonar of 0, which suggest that they do not require a significant cognitive effort from the control flow point of view. In fact, the cognitive load measured using the two EEG features considered in our experiments [the same features used in Medeiros et al. (2021)] showed quite low values. Even so, they were not zero (i.e., they required a cognitive load higher than the control task), and the values obtained for the main unit of C1 were much higher than the value for C2. Table 7 shows the code of the main units of C1 and C2. Both units are very simple and have the minima values of V(g) and CC-Sonar, but C1-main is clearly more complex from the data point of view than C2-main. Additionally, C1-main has more LoC than C2-main. These results raise the following observation, which is consistent with the results obtained for other code regions (see Table 8 further on):

**TABLE 7:** Code of the main units of C1 and C2, showing that even small differences in the code could lead to different average cognitive load measured by EEG.

Program C1, main V(g) = 1 CC-Sonar = 0 / 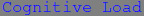 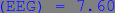	Program C2, main V(g) = 1 CC-Sonar = 0 / 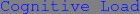 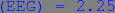
public static void main(String[] args) { int[] sequence = {−7, 1, 5, 2, −4, 3, 0}; int result = getResult(sequence, 2, 4); System.out.println(“Result = ” + result); }	public static void main(String[] args) { System.out.println(getResult(“1234”, “56789”)); }

**TABLE 8:** Detailed results for code regions.

Region	LoC	V(g)	Volume (HVol)	Length (HLth)	Effort (HEff)	Number of operands	Difficulty (HDif)	CC-BCS	CC-Sonar	Number of revisits			Cognitive load (EEG)		
										**All**	**Ordinary**	**Proficient**	**All**	**Ordinary**	**Proficient**
C1.A	4	2	108.4	26	948.7	14	8.75	20	1	7	8	6	5.39	5.27	5.61
C1.B	6	4	153.7	35	2,613.4	17	17	132	4	11	12	10	8.96	9.72	7.71
C1.C	5	1	208.9	43	1,319.3	24	6.32	9	0	9	8	12	7.60	6.64	9.38
C1.AB	10	5	222.9	48	4,557.2	23	20.44	192	5	19	22	15	14.35	14.99	13.33
C2.A	12	4	468.8	90	9,650.5	39	20.58	1,550	4	33	41	27	25.54	21.85	28.77
C2.B	4	1	177.2	41	1,860.6	21	10.5	15	0	13	12	13	6.99	6.48	7.43
C2.C	17	4	666.8	128	21,819.5	66	32.72	3,512	5	98	103	93	53.58	56.32	51.18
C2.D	9	4	390.7	75	6,662.6	36	17.05	150	4	12	12	12	5.59	4.50	6.68
C2.E	3	1	81.7	20	286.1	10	3.5	8	0	5	5	6	2.25	1.43	3.06
C2.C.1	3	2	171.9	38	1,575.7	20	9.17	70	1	19	25	14	8.67	10.16	7.36
C2.C.2	6	2	340.0	68	5,780.0	34	17	196	2	79	84	74	33.37	35.09	31.87
C2.C.3	8	2	195.0	42	2,482.3	20	12.73	165	2	19	21	17	11.54	11.07	11.94
C2.BCD	30	7	1,295.0	224	62,116.5	107	48	6,608	9	114	120	110	66.16	67.30	65.29
C3.A	7	10	438.8	97	9,049.9	45	20.62	36	6	19	19	19	10.16	12.14	7.06
C3.B	4	1	158.1	36	543.55	22	3.44	90	0	5	6	4	1.96	2.25	1.20
C3.C	32	13	1,468.8	276	81,066.6	122	55.19	4,379,438	48	116	90	150	93.64	76.24	116.85
C3.A.1	3	5	171.0	41	1,624.19	19	9.5	20	2	9	9	9	3.88	3.12	4.89
C3.A.2	2	3	112.6	27	914.7	13	8.12	3	2	3	3	2	0.86	0.94	0.75
C3.A.3	2	4	229.3	55	3,583.5	25	15.62	3	2	7	4	10	1.13	1.35	0.98
C3.C.1	29	12	1,338.2	255	68,048.5	113	50.85	625,646	47	76	58	100	84.70	66.40	109.10
C3.C.2	27	11	1,222.8	233	56,675.3	103	46.35	89,390	45	72	55	95	77.91	60.06	101.70
C3.C.3	3	1	166.8	40	1,061.44	20	6.36	12	0	5	8	3	4.41	2.47	6.35
C3.C.4	19	9	583.0	120	13,058.2	48	22.4	9,933	38	42	33	56	43.20	36.25	52.46
C3.C.5	11	6	413.4	86	7,235.07	35	17.5	1,200	24	30	27	34	31.80	27.81	37.11
C3.C.6	9	5	346.1	72	5,191.9	30	15	170	19	21	24	17	20.28	19.43	21.40
C3.C.7	2	3	236.8	51	2,404.8	22	10.15	72	7	16	22	7	7.04	3.39	10.70
C3.C.8	4	2	142.6	33	816.8	14	5.37	12	6	5	5	7	1.65	1.94	1.37
C3.C.9	2	2	48.4	14	254.2	6	5.25	6	6	2	2	1	0.60	0.60	0.60
C3.C.10	4	2	64.7	17	258.9	6	4	12	5	2	2	1	0.70	0.44	0.87
C3.C.11	2	2	48.4	14	254.3	6	5.25	6	5	1	1	1	0.24	0.24	0.24
C3.C.12	4	2	303.1	67	3,214.8	33	10.61	48	4	5	5	4	5.66	3.50	8.91

*RQ4 – Can cognitive load measured by EEG detect small differences in the complexity of code units?*

*Results for small code units with the lowest values of V(g) = 1 and CC-Sonar = 0 show that Cognitive Load (EEG) can discriminate small differences in the actual complexity of the code, particularly complexity related to data structures and number of lines of code that is not well captured by V(g) and CC-Sonar.*

An interesting result in Table 6 is the contradictory values obtained for CC-Sonar and Cognitive Load (EEG) for the units C1.function and C2.function1. In fact, C1.function has a CC-Sonar value of 5 and a Cognitive Load (EEG) of 14.35, while C2.function1 has a lower value of CC-Sonar of 4 but a value of Cognitive Load (EEG) of 25.54, which is much higher than the value measured for C1.function. The same can be observed for V(g), as this metric has the same values of CC-Sonar for these two units.

The use of thresholds of code complexity metrics to refactor code units (i.e., break the unit in two simpler units) has an important problem: it lies in the assumption that the complexity metric is monotonically increasing with respect to programmers’ effort in comprehending the code, which is measured in our study through Cognitive Load (EEG). Obviously, an inversion in the monotonicity of CC-Sonar, such as the one found in the cognitive load results for the units C1.function and C2.function1, is a serious problem. It may introduce unnecessary fragmentation in the software architecture or fail in refactoring highly complex units.

*RQ5 – Do V(g) and CC-Sonar metrics have always a monotonic behavior?*

*Results for C1.function and C2.function1 (and for other code regions; see Table 8) show that V(g) and CC-Sonar metrics do not always show a monotonic behavior.*

Given the very high industrial relevance of metrics such as CC-Sonar and V(g) for code refactoring, it is important to try to understand the reasons why CC-Sonar does not represent well the average cognitive load of programmers while comprehending the code of these two units. Table 9 shows the code of C1.function and C2.function1.

**TABLE 9:** Example of code that caused a divergence between CC-Sonar values and the cognitive load measured by EEG.

C1.function CC-Sonar = 5 / 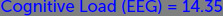	C2.function1 CC-Sonar = 4 / 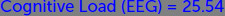
public static int getResult(int[] sequence, int lower, int upper){ int result = 0; if (sequence == null) return result; for (int n : sequence) { if (n > = lower && n < = upper) result++; } return result; }	private static byte[] getInts(String digs) { byte[] result = new byte[digs.length()]; for (int i = 0; i < digs.length(); i++) { char c = digs.charAt(i); if (c < ‘0’ | | c > ‘9’) { throw new IllegalArgumentException(“Invalid string” + c + “at position” + i); } result[digs.length()-1-i] = (byte) (c-‘0’); } return result; }

The mere observation of the code of these two units immediately shows that C2.function1 is clearly more difficult to understand than C1.function, in spite of the values of CC-Sonar and V(g) may suggest the opposite. The following observations help to understand which these metrics, and particularly CC-Sonar, fail in this type of code scenario:

#### 4.2.1. Variables

The use of variables seems to play an important role. This aspect is captured by Halstead metrics but not by V(g) nor CC-Sonar. This example shows that the quantity and complexity of variables (and operands, and parameters) have a clear impact on the difficulty programmers may feel in comprehending code. This seems logical as the existence of more data components (such as in C2.function1) forces the programmers to hold more information in their short-term memory.

#### 4.2.2. Library and external API

The use of secondary aspects of the language (e.g., calls to library functions and external APIs) are not accounted for code metrics. This is, in fact, hard to address because the meaning of such calls may not be evident in the context of the code under analysis, which seems to play a role in the increasing of cognitive load of the participants in our experiment.

#### 4.2.3. Algorithm

CC-Sonar and V(g) metrics do not address the semantics of the program operations. In other words, the algorithm and what it means for the programmer are not captured by those metrics. Handling a program that uses a complex algorithm necessarily puts an additional cognitive load on the programmer, even if the code constructs are simple. The semantics of the algorithm is clearly more complex in C2.function1 than in C1.function, even if the code constructs [relevant for CC-Sonar and V(g)] are very simple.

*RQ6 – Why do V(g) and CC-Sonar metrics deviate considerably from the cognitive load measured using EEG for some code units?*

*Results show that data complexity, the use of libraries and APIs, and the semantics of the algorithms (i.e., aspects that may make the algorithm complex beyond the cyclomatic complexity) are elements that could be included in metrics such as CC-Sonar to capture code complexity in a human perspective in a more accurate way.*

Another evident result in Table 6 is the lack of saturation effect in existing code complexity metrics. That is, there is no upper limit for the values obtained for the code complexity metrics. This is the case of some Halstead metrics and CC-BC (weighting basic control structures) that reach very high values for some units. It is obvious that these very high values of complexity metrics do not have a clear meaning in terms of code complexity perceived by programmers. This was expected, as the complexity perceived by the programmer (or the difficulty in comprehending code) depends on both the programmer (e.g., his/her level of programming expertise) and the code, and the complexity metrics are entirely defined based on code structure and data.

Although metrics are calculated considering only the information available in the source code, most metrics have been defined with the objective (at least partially) of capturing code complexity as perceived by average programmers. For example, the popular metric CC-Sonar has been proposed to overcome the limitations of V(g) in representing code complexity for code refactoring purposes (Campbell, 2017). But the results in Table 6 show that CC-Sonar does not have a saturation effect as well. For example, the unit C2.function2 had CC-Sonar of 9 and an average value of Cognitive Load (EEG) of 66.16, while C3 had CC-Sonar of 55 (a huge value), but the Cognitive Load (EEG) was just 1.8 times higher (123.03) than the value observed for C2.function2.

The saturation effect already observed in the NASA-TLX results is also very evident in the cognitive load measured by EEG and a value in the range of 140 for the cognitive load measured using the two EEG features considered in our experiments [same features used in Medeiros et al. (2021)] seems close to the saturation point for average programmers. In fact, the maximum average value of Cognitive Load (EEG) observed was 141.86 (±33.55) (see Table 5) for proficient programmers trying to comprehend C3. The analysis of the eye tracking data showed that these programmers tried harder to understand this difficult code with V(g) = 22 (i.e., they were engaged during the 10 min of maximum time). The large confidence interval resulted from the fact that the number of proficient programmers was small.

Since the value of CC-Sonar recommended as threshold to refactor a code unit is 15 for most languages (and can go up to 25 for C and C++) (Campbell, 2021), the high value of cognitive load measured by EEG for C2.function2 suggests that for this specific code the CC-Sonar of 9 [and V(g) = 7] does not capture well the difficulties evidenced by participants in understanding program C2 in general and C2.function2 in particular. Note that the average performance of participants in understanding C2 was 2.6 (on a 0 to 6 scale). Even proficient programmers scored only 3.68 in the comprehension of C2 (see Table 5), which means the code C2.function2 was not well understood, despite having a CC-Sonar value substantially lower than the threshold of 15.

*RQ7 – Code units with values of V(g) and CC-Sonar metrics much lower than the threshold values used for code refactoring should correspond to code units that are easily understood by the average programmer?*

*Results show that values of CC-Sonar [and V(g)] much lower than the value recommend as threshold for code refactoring do not guarantee that programmers easily understand the code unit, suggesting that other elements in the code (in addition to control flow complexity) may cause higher cognitive load to the programmers and reduce their performance in comprehending the code.*

Table 8 shows the results for the code regions defined in the three programs. In addition to all the code metrics already used in previous tables and cognitive complexity (EEG) used as reference, Table 8 also shows number of revisits to each code region, which is a metric based on the participants’ code reading behavior (i.e., not in the code). The calculation of code metrics such as CC-BCS and CC-Sonar for the inner code regions of each unit (e.g., a While structure that is inside two For loops) was done assuming that such code region was a code unit *per se* and mimicked the same cyclomatic conditions where the code region is inserted in the program unit.

The goal of the analysis of the detailed results shown in Table 8 is to help identifying specific code regions causing high programmers’ cognitive load, to identify examples of code and data structures that are responsible for increased effort in comprehending code. Note that the code regions are used only for analysis purposes and were not visible in the code of the programs during the code comprehension tasks.

A high number of revisits to a given code region could be interpreted as difficulty in understanding the code of the region, which takes the programmer to look at that code many times. Another possible interpretation is that the programmer is meticulous and was really confirming the meaning of the code (possibly because that code is related to other regions). Both interpretations lead to the conclusion that a high number of revisits to a given code region is associated with high cognitive load, which may indicate difficulty of the programmers in comprehending that code snippet. In fact, the Spearman correlation coefficient (*r*_*s*_) calculated for number of revisits (all programmers) and the cognitive load measured by EEG (all programs) result in *r*_*s*_ = 0.963 with a corresponding *p* < 0.0001, which indicates a very high correlation.

*RQ8 – Is the number of revisits to the code regions of a program a reliable indication of the programmers’ cognitive load required to understand the code of the different code regions?*

*The strong positive correlation observed between the number of revisits to a given code region and the cognitive load measured using EEG* (r = *0.963 with* p *< 0.0001) suggests that the measurement of programmers’ cognitive load in program comprehension tasks can be achieved using a simple and non-intrusive eye tracker (to calculate the number of revisits to each code region of the program) instead of a complex EEG setup. Furthermore, while EEG can be used only in controlled experiments, the low intrusiveness of eye trackers opens the possibility of using this approach to measure programmers’ cognitive load in real software development environments.*

It is worth mentioning that this result is obviously dependent on the algorithm used for the division of the code into code regions. In our study, we used an algorithm (presented in section “3.2 Programs and code regions of analysis”) that tries to mimic the way the average programmer is expected to work out the code to fully understand it.

For space reasons, the following paragraphs focus only on the analysis of CC-Sonar and cognitive load (EEG) values for the different code regions, with the main goal of identifying code scenarios where CC-Sonar does not capture well the effort needed (i.e., cognitive load) by programmers in understanding the code. We see this as an important step towards the proposal of new variants of CC-Sonar metric that match the programmers’ cognitive load in a more effective way.

Table 8 shows that there are many code regions where the CC-Sonar value does not correspond to the cognitive load measured using EEG or breaks the monotonicity of the CC-Sonar metric. For example, the regions C2.C and C2.D, although having similar values of CC-Sonar (5 and 4, respectively) show very different values of cognitive load (53.58 and 5.59, respectively). An important difference between the code of these two regions is related to the data structures, number of variables and parameters, which are much more complex in the case of C2.C. This is well captured by the five Halstead metrics that show much higher values for C2.C than for C2.D. This effect [i.e., high values of cognitive load (EEG) for code regions with high values of Halstead metrics] can be observed in many code regions in Table 8. There is in fact a high positive correlation between Effort (HEff) and cognitive load (EEG) with a Spearman correlation coefficient value of *r*_*s*_ = 0.901 with a corresponding *p* < 0.0001. All the other Halstead metrics (Halstead, 1977) correlates with cognitive load (EEG) (the one with lower *r*_*s*_ had *r*_*s*_ = 0.85 with *p* < 0.0001). A possible reason for not having even higher Spearman coefficient values is because the physiologic measurement of cognitive load (EEG) saturates in the high values, which is not the case of Halstead metrics.

In contrast with Halstead metrics, the correlation between the values measured for CC-Sonar and cognitive load (EEG) for the 29 code regions show a Spearman correlation coefficient of *r*_*s*_ = 0.513, with a corresponding *p* = 0.00316. Although there is a positive correlation, the fact that the coefficient *r*_*s*_ is lower than the one observed for the Halstead metrics suggests that the code regions with high data complexity are causing such deviation.

*RQ9 – Is CC-Sonar measuring accurately the programmers’ cognitive load related to the data complexity of code units?*

*Results show that code regions with high data complexity, as captured by Halstead metrics, correspond to high values of participants’ cognitive load (EEG), showing that data complexity is an important cause of possible difficulties in comprehending code that is normally not captured by the CC-Sonar metric for the same code regions. This is a strong indication that CC-Sonar should be complemented with some “flavor” of Halstead metrics.*

Code regions C2.C.2 and C2.C.3 also show a clear discrepancy between the values obtained for CC-Sonar and code complexity (EEG), as low values of CC-Sonar correspond to relatively high values of cognitive load. However, in this case the data complexity is not the only reason for the high participants’ cognitive load, as happened in region C2.C, since the values of Halstead metrics are moderate. Table 10 shows the code snippets for these two regions (and C2.C.1 to provide context). Although the structure of the code is rather simple in both C2.C.2 and C2.C.3 (hence CC-Sonar is low), the real meaning (i.e., the algorithm) of the instructions inside the for cycle (C2.C.2) and the while cycle (C2.C.3) is not obvious.

**TABLE 10:** Examples of code regions with low CC-Sonar and high cognitive load (EEG).

**C2.C.1** **CC-Sonar = 1** 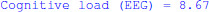	for (int rightPos = 0; rightPos < right.length; rightPos++) { byte rightDigit = right[rightPos]; byte temp = 0;
**C2.C.2** **CC-Sonar = 2** 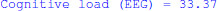	for (int leftPos = 0; leftPos < left.length; leftPos++) { temp + = result[leftPos + rightPos]; temp + = rightDigit * left[leftPos]; result[leftPos + rightPos] = (byte) (temp % 10); temp / = 10; }
**C2.C.3** **CC-Sonar = 2** 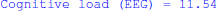	int destPos = rightPos + left.length; while (temp ! = 0) { temp + = result[destPos] & 0xFFFFFFFFL; result[destPos] = (byte) (temp % 10); temp / = 10; destPos++; } }

A final example of strong discrepancy between CC-Sonar and cognitive load (EEG), but this time following the opposite direction as the one shown in Table 10, is the result observed for the regions C3.C.8, C3.C.9, C3.C.10, and C3.C.11. In this case we observe a reasonably high value of CC-Sonar (5 or 6) but the corresponding values of cognitive load (EEG) are rather low.

Table 11 shows the code of these regions. The reason why CC-Sonar is relatively high is because the code of these regions is nested inside several for loops. As CC-Sonar increases 1 point for each nesting level of control flow breaking structures, the metric shows a relatively high value. Since the code of these regions is very simple and is not dependent (through the variables) of the outer for loops, the programmers easily understand these regions and the measured cognitive load (EEG) is rather low.

**TABLE 11:** Examples of code regions with relatively high CC-Sonar and very low cognitive load (EEG).

**C3.C.8** ** CC-Sonar = 6** 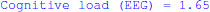	if (c = = 0) { 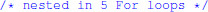 mb = b; break; }
**C3.C.9** **CC-Sonar = 6** 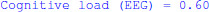	if (c < mc) 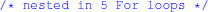 mc = c;
**C3.C.10** **CC-Sonar = 5** 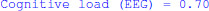	if (b = = 0) { 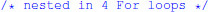 ma = a; break; }
**C3.C.11** **CC-Sonar = 5** 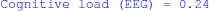	if (b < mb) 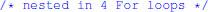 mb = b;

The reason why the values of CC-Sonar metric are relatively high for the code regions shown in Table 11 is because the CC-Sonar metric increments 1 for each nested level in the code. However, high levels of nested code do not always correspond to high comprehension effort from the programmers.

*RQ10 – Is the depth of nesting of control flow breaking structures always responsible for the increase in the* programmers’ *difficulty in understanding the code, as proposed by CC-Sonar metric?*

*Results show that the depth of nesting of control flow breaking structures, although increasing the value of CC-Sonar, does not necessarily lead to high levels of participants’ cognitive load. This is particularly the case when the variables and operands involved in the execution of the nested flow breaking structures are not used in the outer loops.*

### 4.3. Guidelines to improve code complexity metrics

The improvement of existing code complexity metrics should be understood (in the context of this study) as the conjunction of three interdependent goals:

a) Assure that the scores provided by code metrics are aligned with the average programmers’ perception of code complexity.
b) Avoid monotonicity failures in the metrics (i.e., an increase in the metric score should not correspond to a decrease in the programmers’ perception of code complexity, and vice versa).
c) Assure that scores thresholds recommended for code refactoring are realistic. Although metrics are used for many other purposes in addition to refactoring, the importance of refactoring in the software industry makes the selection of the right threshold a relevant goal *per se*.

The next points summarize our proposal of guidelines to improve code complexity metrics such as CC-Sonar and V(g). For space reasons, we just propose the guidelines as a spotlight pointing to possible future research directions, considering that such guidelines are supported by the observations previously presented in this section (paragraphs in italic):

#### 4.3.1. Saturation

Code complexity metrics such as CC-Sonar and V(g) should include a scale saturation effect in the scores, as experimental results show a clear saturation in the programmers’ perception of code complexity. The human notion of software unit complexity saturates at a given point and metrics should reproduce that for the average programmer.

#### 4.3.2. Nonlinearity and threshold scores

Programmers’ cognitive load measured using EEG suggests a nonlinear perception of code complexity. This must be studied in detail to understand how the complexity perception grows and allows a solid justification for the scores used as threshold for code refactoring. The quest for the optimal average saturation threshold (very relevant for code refactoring) and/or the investigation of personalized methods to identify complexity saturation points for individual programmers are important research lines where neuroscience can contribute to improve software engineering practices.

#### 4.3.3. Data complexity

Code complexity metrics based on a cyclomatic complexity perspective [such as CC-Sonar and V(g)] should include data complexity elements as the ones used by Halstead effort metric. Experimental results clearly show that data complexity (including the number of variables and parameters) plays an important role in the programmers’ perception of code complexity.

#### 4.3.4. Algorithm complexity

The idea that the algorithm complexity in a software unit can be measured by cyclomatic complexity clearly has strong limitations. The semantics of sequential instructions (especially data movement instructions) can significantly increase the programmers’ perception of complexity. Existing metrics must incorporate other elements of algorithm complexity, in addition to cyclomatic complexity.

#### 4.3.5. Context complexity

The use of libraries, APIs, services, methods, etc. by a software unit seems to contribute more for the programmers’ perception of complexity than just as mere flow breaking elements, as they are often treated by existing metrics. The general notion of fan-out (number of other functions, methods, etc., called by a given unit) embeds additional context complexity that must be incorporated in existing metrics.

#### 4.3.6. Depth of nesting

The experimental results show that the number of statement blocks that are nested due to the use of control structures (loops and branches) do not necessarily increases the difficulty in comprehending the inner blocks, especially when the execution of the code in the inner block is independent from the outer loops. Existing metrics should take that into consideration and do not assume that nesting always increase the code complexity.

### 4.4. Limitations and threats to validity

Studies involving human being performance in controlled experiments usually have limitations and threats to validity in the different aspects. However, we applied several measures to mitigate potential threats in the following elements.

#### 4.4.1. Construct validity

The validity here stands for measures taken to mitigate the risks that threaten data reliability. First, this study uses EEG as the reference for the programmers’ cognitive load measurement, which can be subject to various artifacts, such as ocular (eye-blink or eye movement), cardiac artifacts, and environmental effects. However, we followed a rigorous and well-established pre-processing pipeline to ensure a reliable neural signal analysis.

Second, the codes used in the comprehension tasks might not perfectly represent real-life problems in software companies, although exhibiting different complexities. The limitation here is the time constraints of a controlled experiment, which results in relatively small programs. However, since the goal is to estimate the cognitive load associated with comprehending different code regions (with different complexities), the effect of small programs is relatively insignificant. Moreover, using these small programs, we illustrated a wide range of practical examples where code complexity metrics fail to express the cognitive load of developers.

#### 4.4.2. Internal validity

This validity deals with the measures taken to mitigate the limitations and threats of data used. The data was acquired in typical controlled experiment environments, which may put constraints on the participants’ performance, and induce the feeling of being monitored or under evaluation. However, we assured all participants that their code comprehension performance would be neither judged nor evaluated under any circumstances. Nonetheless, the feeling of being monitored or observed varies among participants, and it is impossible to eliminate it entirely.

#### 4.4.3. External validity

This validity deals with mitigating the risks of results’ generalizability.

First, although the results of this study can be replicated and generalized, one of the obvious limitations is the limited number of participants recruited (i.e., 27 participants), given the personalized nature of the neural measurement. We attempted to overcome the limitation by recruiting two levels of JAVA expertise and using various code complexities to achieve a reasonable level of generalizability.

Despite these threats and limitations, this study rigorously evaluated state-of-the-art code complexity metrics using a comprehensive set of examples and cases and compared them with the complexity perceived by humans using cognitive load measurement from a reliable well-established source (i.e., EEG).

## 5. Conclusion

This article discusses the results of a controlled experiment involving 27 programmers performing code comprehension tasks and shows that popular code complexity metrics fail considerably in capturing code complexity as perceived by programmers. The reported experiment used programmers’ cognitive load measured by EEG as the reference to evaluate a comprehensive set of existing code complexity metrics in terms of their ability to capture the difficulty programmers may feel in comprehending code. The metrics evaluated include classic McCabe and Halstead metrics, cognitive complexity metrics based on scored code constructs, programmers’ behavior metrics such as reading time and number of revisits to specific code regions, and state-of-the-art code complexity metrics such as the metric provided by SonarSource tools (named in the paper as CC-Sonar).

The experiment used additional survey-oriented methods based on NASA-TLX to assess the subjective perception of participants in terms of code complexity. Results showed that the programmers’ cognitive load measured using EEG correlates (*r*_*s*_ = 0.829, with *p* < 0.0001) with the subjective perception of code complexity assessed by NASA-TLX, supporting our assumption that cognitive load measured through EEG in our experiment represents well the difficulty programmers may feel in comprehending code and constitutes a good yardstick to compare complexity metric.

The results of the evaluation of the different metrics were discussed at the level of the entire programs used in the code comprehension tasks (the experiments used three programs), code units (functions) of each program, and small code regions used in the analysis to help identify the actual code elements that cause high values of programmers’ cognitive load, which correspond to code areas that were perceived as complex by the programmers.

Given the relevance for the software industry of metrics such as V(g) and CC-Sonar, the discussion of the results was particularly focused on the evaluation of such metrics. In particular, the paper presented a comprehensive set of examples (illustrated using the actual code) in which V(g) and CC-Sonar seemed to fail in capturing code complexity as perceived by programmers, discussing in detail possible causes and possible solutions to improve the accuracy of current code complexity metrics.

The article ends with the proposal of a set of guidelines encompassing both concrete proposals and research directions to improve existing code complexity metrics, particularly metrics such as CC-Sonar.

## Data availability statement

The original contributions presented in this study are included in the article/Supplementary material, further inquiries can be directed to the corresponding author.

## Ethics statement

The studies involving human participants were reviewed and approved by the Ethics Committee of the Faculty of Medicine of the University of Coimbra, https://www.uc.pt/fmuc/orgaosconsultivos/comissaoetica. The patients/participants provided their written informed consent to participate in this study.

## Author contributions

GH: analysis, writing, and results and tables. HH: writing, repository preparation, and reviewing. JD: experiment design, analysis, writing, and reviewing. JM: analysis, writing, data curation, repository preparation, EEG processing pipeline, and reviewing. RC: EEG processing pipeline and data curation. CL: supervision. CT: supervision and EEG processing pipeline. JC: experiment design, data acquisition, and EEG resources. MC: neuroscience supervision, EEG resources, and experiment design. PC: conceptualization, methodology, funding acquisition, and reviewing. HM: conceptualization, methodology, funding acquisition, writing, and reviewing. All authors contributed to the article and approved the submitted version.

## Footnotes

1
shorturl.at/DHT79
